# To Be Bicultural Is to Be Friends? Associating Early Adolescents' Cultural Identities and Behaviors With Friendship Networks in Minority‐Only Schools

**DOI:** 10.1002/jcop.70081

**Published:** 2026-01-07

**Authors:** Jessie Hillekens, Tobias H. Stark, Karen Phalet

**Affiliations:** ^1^ Department of Developmental Psychology Tilburg University Tilburg The Netherlands; ^2^ Center for Social and Cultural Psychology University of Leuven Leuven Belgium; ^3^ Department of Interdisciplinary Social Science Utrecht University Utrecht The Netherlands

**Keywords:** acculturation, adolescent development, cultural behavior, cultural identity, friendship networks, minority‐only schools

## Abstract

Many ethnic minoritized adolescents attend minority‐only schools with no ethnic majoritized classmates, but we know little about their acculturation and friendships in such settings. This study examined how early adolescents' heritage and mainstream cultural identities and behaviors associated with friendship ties in minority‐only class networks. Early adolescents (*N* = 146, *M*_age_ = 11.24, 54.4% boys) in minority‐only primary schools reported their heritage and mainstream cultural identities and behaviors and completed a sociometric measure of their friendship networks. Exponential Random Graph Models revealed that stronger heritage cultural identities and behaviors were associated with more friendships in class. Whereas mainstream cultural behaviors were linked to more friendships, stronger mainstream cultural identities were associated with fewer friendships, however. Early adolescents' cultural orientations were linked to friendship ties in minority‐only classes, yet associations differed between cultural identities and behaviors. Our findings highlight the need for a more nuanced approach of acculturation in early adolescence.

## 1 Introduction

European societies are plagued by ethnic segregation in the sense that ethnic minoritized and ethnic majoritized adolescents are separated into different social spaces, activities, and networks from an early age (Musterd 2016). Ethnic segregation is a process used by the dominant group to maintain their social status, inequity, and structural racism, separating youth into more privileged versus more marginalized communities. It is not only present in society at large, such as in neighborhoods and cities, but also in the school system. Accordingly, ethnic segregation channels ethnic minoritized adolescents into different schools and classrooms from their majoritized peers, resulting in school environments where they mainly encounter other ethnic minoritized peers (Holmlund and Öckert 2021). In what we call minority‐only schools, all pupils have an ethnic minoritized background. Growing up in such school contexts leads to specific schooling experiences for large numbers of minoritized youth. On the one hand, minority‐only schools provide unique environments for intercultural learning and interactions and peers are often more welcoming toward the diverse cultural backgrounds of students in these schools (Hillekens et al. 2019). On the other hand, these schools can result in unequal schooling experiences that perpetuate systemic inequalities in educational and later attainments (Musterd 2016; Holmlund and Öckert 2021). Despite it is the lived reality of many minoritized adolescents, research has not yet systematically examined the specific experiences of adolescents attending minority‐only schools because such schools have typically been mixed together with schools with a different ethnic composition.

To address this gap, this research studies the link between early adolescents' acculturation orientations and their friendship ties (who befriends whom?) in minority‐only schools. In line with bidimensional acculturation frameworks (Berry 1997; Ryder et al. 2000), we define acculturation orientations as minoritized adolescents' generalized commitments toward the mainstream culture of society and the heritage cultures of their families' countries of origin (Bourhis et al. 1997). By combining an intergroup relations approach of acculturation (Brown and Zagefka 2011; Phalet and Baysu 2020) with an ecological‐systems theory approach (Bronfenbrenner 2005), we conceive of acculturation processes as part of adolescent development in the peer context. While adolescents share the mainstream culture with all peers in minority‐only schools, they also share their distinct heritage culture with co‐ethnic peers only. Most minoritized adolescents develop strong orientations toward both cultures (Verkuyten et al. 2019), which might be associated with their friendships in minority‐only schools.

Although acculturation research generally focuses on acculturation orientations more broadly, cultural orientations vary between different acculturation domains, such as cultural values, identities, and behaviors (Schwartz et al. 2010). This study extends prior work by taking a domain‐specific approach toward acculturation as different acculturation domains might have different links to adolescents' friendships. We focused specifically on cultural identities and behaviors given the age of our sample (10–13 years old), since reflecting on cultural values requires abstract reasoning that only develops later in adolescence (Knafo‐Noam et al. 2023). Using sociometric data on early adolescents' friendship networks, we therefore examined whether cultural identities and behaviors were associated with friendship ties in minority‐only school classes in Molenbeek, Brussels, Belgium.

### 1.1 Acculturation Orientations and Friendships in Minority‐Only Schools

Minoritized early adolescents who grow up in minority‐only schools likely have very different acculturation experiences than those who grow up in schools with a different ethnic composition. Building on an intergroup relations approach of acculturation (Brown and Zagefka 2011; Phalet and Baysu 2020), minoritized adolescents' acculturation orientations are contingent on cultural expectations in their social environments, such as in minority‐only schools. Additionally, and in line with an ecological‐systems theory approach (Bronfenbrenner 2005), adolescent development takes place within distinct subsystems of relationships, with peer relations in school being a subsystem of critical importance in early adolescence. We therefore comprehend acculturation processes as part of adolescent development situated in the peer context (Schachner et al. 2017; Titzmann and Lee 2018). Yet, peer relations might be very different in minority‐only schools compared to schools with a different ethnic composition so that adolescents' acculturation experiences also differ. Early adolescents in minority‐only schools may encounter peers from many different origins, yet they do not encounter any ethnic majoritized peers. This raises the question how they co‐construct their heritage and mainstream cultural orientations among peers.

Previous research has highlighted the interplay between peer relations and the development of adolescents' orientations toward both heritage and mainstream cultures. For example, stronger co‐ethnic friendship networks were cross‐sectionally associated with stronger heritage orientations and variably weaker mainstream cultural orientations (Hällsten et al. 2018); and stronger mainstream orientations were associated with better sociocultural adjustment including more friendships with ethnic majoritized peers in class (Schachner et al. 2018). Most prior longitudinal research has examined peer relations as a predictor of acculturation orientations over time. Thus, acculturation orientations are informed by friendships with ethnic majoritized youth (Hillekens et al. unpublished manuscript; Jasini et al. 2023) and peer norms in schools over time (Hillekens et al. 2019); and adolescents with more diverse friendships reported more exploration of their heritage cultural identity over time (Rivas‐Drake et al. 2017). Still, it can be argued that youth's cultural orientations can also shape their peer relations as explained in the next sections.

Acculturation theory generally distinguishes between psychological (“feeling well”) and sociocultural (“doing well”) adjustment outcomes of acculturation processes (Arends‐Tóth and van de Vijver 2006). From this perspective, friendships can be argued to be a specific form of sociocultural adjustment (see also Schachner et al. 2018). Friendships are a key indicator of the formation and maintenance of positive social relationships with peers and are a critical protective factor in adolescent development. Due to high levels of social support and trust among friends (Reynolds 2007), friendships satisfy many social and psychological needs, such as the need to belong, and benefit adolescents' self‐esteem (Stets et al. 2021). Consequently, friendships have critical implications for adolescents' well‐being (Li and Goldman 2024; Meyer et al. 2023), health, and quality of life (Stets et al. 2021). It is therefore especially relevant to investigate which factors are associated with friendships since it might be a way to also promote minoritized adolescents' wider well‐being. Given that the benefits of friendships can be expected for same‐ and cross‐ethnic friendships alike, we focus on general likelihood of friendships as a critical sociocultural outcome.

Despite the aforementioned, friendships have received less attention as an outcome in acculturation research—although acculturation orientations and peer relations are likely bidirectionally related. In line with social structure theories (Blau 1977; Stauder 2014), social relationships are shaped by immediate social environments—such as classes—and the opportunities for contact and interaction that they provide. For example, early adolescents in minority‐only schools are restricted in their opportunities to form friendships with ethnic majoritized peers due to the absence of these peers in their schools. Within these immediate social environments, adolescents are more likely to be attracted to peers that they perceive as similar to themselves, as proposed by Byrne's (1971) similarity‐attraction hypothesis. Thus, friendships within minority‐only classes might be more likely to occur between early adolescents who share similar acculturation orientations. In line with this, some prior longitudinal evidence suggests that adolescents also form friendships based on similar heritage cultural orientations (Leszczensky and Pink 2019). Still, little research has focused on associations between acculturation orientations and friendship ties (who befriends whom) in minority‐only schools—which we examined in this study.

Arguably, peers' cultural expectations are different in minority‐only schools compared to schools with a different ethnic composition. On the one hand, adolescents value peers' heritage cultures more in schools with larger shares of minoritized pupils like in minority‐only schools (Hillekens et al. 2019; Rock et al. 2011). On the other hand, the mainstream culture is the shared culture among all adolescents in school. Minoritized youth in minority‐only schools together challenge narrow definitions of who belong to the mainstream culture and redefine the boundaries of the mainstream cultural group (Wood 2022). It is therefore likely that the mainstream culture is equally promoted among peers in minority‐only schools compared to schools with a different ethnic composition (Hillekens et al. 2019), although its definition might be more inclusive (Gharaei et al. 2018). It is therefore likely that both heritage and mainstream cultural orientations are considered valuable in minority‐only schools and might be associated with more friendships. However, little research has examined these acculturation processes in these schools.

### 1.2 Cultural Identities and Cultural Behavior as Distinct Acculturation Domains

We distinguished between cultural identities and cultural behaviors as distinct acculturation domains (Schwartz et al. 2010). According to identity homophily theory (Stets et al. 2021), identities are a binding factor between people that define the way in which they interact with each other so that identities might shape, and be formed by, friendships. Some identities are hereby more prominent or central than others depending on the relative importance of the identity for individuals in particular contexts (Stets et al. 2021). Arguably, cultural identities are one of the most central identities in minority‐only schools so that attaching similar importance to cultural identities might be a critical driver for minoritized adolescents to become friends and vice versa. Importantly, identities and behaviors are linked because they share similar meanings: behavior verifies people's identities and ensures that identities align with contextual expectations (Stets et al. 2021). Accordingly, it can be argued that cultural identities signal adolescents' cultural attachments, whereas cultural behaviors relate to their cultural performances. Cultural identities and behaviors might be congruent or incongruent with each other; and may align or de‐align with cultural expectations of peers (Van Laar et al. 2014). It is therefore likely that both heritage and mainstream cultural identities and behaviors are associated with early adolescents' friendship ties in minority‐only schools.

There is indeed some evidence bidirectionally linking cultural identities to friendship ties. Adolescents who have more co‐ethnic friends showed a more central ethnic identity over time (Douglass et al. 2017) and talking about cultural identities with friends reinforced adolescents' own cultural identities, both for heritage and mainstream cultures (Vietze et al. 2019). Closer to the present study, there is also some research that examines associations between cultural identities and adolescents' friendship networks. For example, studies using longitudinal social network approaches in Germany (Jugert et al. 2020) and the United States (Rivas‐Drake et al. 2017) showed that adolescents' heritage cultural identities are influenced by their friends, so that adolescents' heritage cultural identities become more similar to those of their friends over time. Additionally, a study in Germany (Leszczensky and Pink 2019) also showed that adolescents select their friends based on heritage cultural identities, so that adolescents befriended peers more easily when they shared strong heritage cultural identities. These studies combined thus demonstrated that there seems to be a bidirectional interplay between heritage cultural identities and adolescents' friendship networks.

Although prior work has predominantly examined the interplay between friendship networks and adolescents' heritage cultural identities, relatively little research has examined the role of mainstream cultural identities. One longitudinal study showed that minoritized adolescents' mainstream cultural identities did not predict, and were not influenced by, their friendships with ethnic majoritized peers (Leszczensky et al. 2016). Another study showed that minoritized adolescents who nominated more ethnic majoritized peers as friends reported stronger mainstream cultural identities over time, but friendships with co‐ethnic peers were unrelated (Munniksma et al. 2015). Moreover, both friendship nominations of ethnic majoritized and co‐ethnic minoritized peers were not predictive of heritage cultural identities over time (Munniksma et al. 2015). Focusing specifically on the formation of friendship ties among minoritized adolescents, Jugert et al. (2017) showed that minoritized adolescents with strong mainstream cultural identities are more often friends with minoritized peers that shared similar mainstream cultural identities. However, this study categorized adolescents into subgroups depending on their position on a unidimensional scale ranging from completely identified with the mainstream culture to completely identified with the heritage culture. Although this study provides indications that mainstream cultural identities might also be associated with minoritized early adolescents' friendship networks, no study to date has examined associations between heritage and mainstream cultural identities and friendship networks among minoritized early adolescents simultaneously.

Even fewer studies have examined cultural behaviors in relation to early adolescents' friendship networks. Still, it is likely that not only cultural identities, but also cultural behaviors play a role. Adolescents are more often friends with peers who are less culturally distant from themselves in terms of their cultural norms and practices (Schachner et al. 2015) and several specific cultural behaviors have been linked to adolescent friendships. For example, qualitative work showed that limited mainstream language proficiency hindered minoritized youth from befriending majoritized peers (Hsin‐Chun Tsai 2006) and that bilingual peers help adolescents who have just migrated to develop their language skills more quickly (Carhill‐Poza 2021). Similarly, food serves as an important connector in social relationships, including friendships. For example, the qualitative work of Julier (2013) showed that sharing a meal can be an important social event that signals validation and social connection. Although these studies on language use and food practices give first indications that cultural behaviors might be relevant in friendships, cultural behaviors can be argued to be much broader than language usage and food practices alone and also include holidays, contact preferences, or specific cultural practices or traditions. Yet, no studies have examined associations between adolescents' cultural identities and behaviors and their friendship networks simultaneously.

### 1.3 The Present Study

This study zooms in on minority‐only schools in Molenbeek, Brussels, Belgium. Molenbeek is a municipality in Brussels that is characterized by strong ethnic segregation and high levels of poverty. Molenbeek used to be a rich industrial area until the Second World War. However, the economic crisis around the time caused many companies to close or leave the area, leading to the ethnic majoritized Belgian working‐class to leave as well. Around the same time, guest workers that were attracted from rural areas of Morocco and Turkey moved in, leading to the ethnic segregation there is nowadays. Around the 70s and 80s, the local government decided to conduct clearance work for the extension of the metro that led to a strong decay of houses and destruction of the living area (Municipality of Molenbeek n.d.). The poverty that resulted from this is still present to this day: compared to other areas of Brussels and Belgium at large, Molenbeek knows double the amount of people dependent on social security and higher unemployment rates, and there are more children who are at least 2 years behind in educational levels (Brussels Institute for Statistics and Analysis 2023).

Using sociometric data on early adolescents' friendship networks, we examined whether cultural identities and behaviors were associated with friendship ties in minority‐only school classes in the last 2 years of primary schools in Molenbeek. We use sociometric data on early adolescents' friendship networks because network data provides information about (the absence of) friendships between all pupils in class and allows to take structural relational characteristics (e.g., reciprocity) into account (Leszczensky and Stark 2019). Moreover, it allows to ask early adolescents about their actual acculturation orientations and link it to their friendship networks, rather than focusing on perceived acculturation orientations of their friends (Leszczensky and Stark 2019). Importantly, adolescents more often befriend peers with a similar ethnicity (Campigotto et al. 2022; Smith et al. 2016), religion (Campigotto et al. 2022; Leszczensky and Pink 2017; Simsek et al. 2022), gender (Mehta and Strough 2009), and age (Völker 2022). We therefore took these factors into account to estimate the net association between acculturation orientations and early adolescents' friendship networks. We hypothesized that stronger mainstream and heritage cultural orientations would be associated with more friendships in minority‐only classes; and we explored potential differences in the role of heritage and mainstream cultural identifications and behaviors as distinct acculturation domains.

## 2 Methods

### 2.1 Participants and Procedure

The data were part of a cross‐sectional study in four Dutch‐speaking primary schools in Molenbeek, Brussels, Belgium. Schools were recruited with the help of an alderman of the municipality and early adolescents in the last 2 years of primary school were sampled. Active consent was obtained from school principals and teachers, while passive consent was obtained from parents and assent was obtained from pupils. The use of passive parental consent was approved by the institutional ethical review board on the grounds that the study involved minimal risks, that parents received clear information and an opportunity to opt out, and that active consent significantly reduces response rates and leads to systemic bias in the sample (Liu et al. 2017). Assent was obtained from pupils through an age‐appropriate explanation of the study, emphasizing repeatedly that their participation was voluntary and that they could stop at any moment without consequences. To minimize possible pressure from teachers, the data were collected by the researchers. Researchers walked around the classroom so that pupils could tell them personally that they wanted to opt out, thereby reducing potential group or teacher pressure.

After obtaining consent from all respective parties, 146 out of 159 eligible early adolescents in 10 classes participated in this study (response rate: 91.8%). Early adolescents were on average 11.24 years old (SD = 0.83, range 10–13 years) and approximately half were girls (45.6% vs. 54.4% boys^1^). All early adolescents had an ethnic minoritized background with the majority having a Moroccan origin (58.5%). The families of those with another cultural background originated from 20 different countries across the world—with most countries being represented by only one early adolescent. Countries that were more frequently represented were Pakistan (*n* = 6), Turkey (*n* = 5), Congo (*n* = 2), Guinea (*n* = 2), and Ghana (*n* = 2). Eleven early adolescents had more than one country of origin. Moreover, early adolescents reported an average of 1.34 (SD = 0.87) identifications with a cultural group other than Belgian, with 20.3% identifying with two or more cultural groups and a maximum of six identifications. Most early adolescents were second or later migration generations, with only 15.6% being first generation and most of them were Muslims (77.4%). The sample was highly multilingual: Early adolescents spoke on average 1.88 other languages than Dutch at home (SD = 0.89, range 0–5), with a total of 17 different languages including French (76.2%), Arabic (50.3%), and Berber (15.6%) as most common ones. Early adolescents filled out questionnaires during class hours in the presence of trained researchers. They first received collective instructions about, and filled out, the sociometric measure to assess their friendships, and afterwards they completed the rest of the questionnaire (including questions about acculturation orientations) at their own pace. Wording of questionnaires was simplified, visual aids were used (e.g., boxes of Likert scales were of increasing size with increasing values), and help was provided to assist early adolescents in filling out the questionnaires.

### 2.2 Measures

*Friendship networks* were assessed using a sociometric measure of early adolescents' best friends in class (Phalet et al. 2018). Early adolescents received a class list (*N* = 12–20 pupils per class) and indicated who they considered to be their best friends in class. They could list as many classmates as they wanted.

*Acculturation orientations:* Early adolescents first reported on their mainstream cultural behaviors and identities, followed by their heritage cultural behaviors and identities. Items examining heritage cultural orientations were preceded by a question asking early adolescents to select the cultural group they felt most belonging to besides Belgian. They were then asked to think about this cultural group when answering the questions that followed.

*Cultural behaviors* were measured with three items for mainstream and heritage cultures each on a scale from 1 (*Completely disagree*) to 5 (*Completely agree*; Brown et al. 2013; Celeste et al. unpublished manuscript). Items examined different types of cultural behaviors (i.e., eating food, celebrating holidays, and preference for contact), such as “I enjoy eating Belgian food” for mainstream cultural behavior and “I enjoy eating food from my cultural group” for heritage cultural behavior. We excluded a fourth item assessing language use in both scales to improve reliability because it did not load appropriately onto the factor. Although removing this item improved the internal consistency for heritage cultural behaviors to an acceptable level (*α* = 0.70), it remained rather low for mainstream cultural behaviors (*α* = 0.59). We refer to the limitations section for a more elaborate discussion.

*Cultural identities* were examined with four items for mainstream and heritage cultures each on a scale from 1 (*Completely disagree*) to 5 (*Completely agree*; Brown et al. 2013). Example items include “How proud are you to be Belgian?” for mainstream cultural identities and “How proud are you to be from your cultural group?” for heritage cultural identities (*α* = 0.92 for mainstream cultural behaviors and *α* = 0.90 for heritage cultural behaviors).

*Control variables* included gender (0 = boys, 1 = girls), age, religion (0 = Muslim vs. 1 = non‐Muslim), and Moroccan origin (0 = non‐Moroccan origin vs. 1 = Moroccan origin). Given the small numbers of early adolescents per country of origin, we could not further distinguish other ethnic groups. Early adolescents were classified as having a Moroccan origin when they or (one of) their parents were born in Morocco and/or when they chose Moroccan as the group they felt most belonging to besides Belgian (see above).

### 2.3 Positionality Statement

All authors are majoritized white scholars from the Netherlands, Belgium, and Germany affiliated to Dutch and Belgian universities. All authors have personal experience with negotiating acculturation orientations having lived and worked outside of their country of birth as international scholars. We are aware that our backgrounds are privileged, as we do not share the experience of being racialized and growing up in highly segregated contexts like the early adolescents in our study.

### 2.4 Analytic Plan

We used exponential random graph models (ERGMs) using the ERGM package (Handcock et al. 2018; Hunter et al. 2008) in R version 4.3.0 to predict friendship ties between early adolescents from their mainstream and heritage cultural behaviors and identities. ERGMs are a type of social network analysis that can be used to predict the probability that two early adolescents in a network (i.e., a pair of nodes) will have a friendship between them (i.e., a tie) based on certain characteristics (i.e., attributes) at the individual or dyadic level. We used mainstream and heritage cultural behavior and identities as well as the control variables gender, age, religion, and Moroccan origin as predictors of outgoing nominations (e.g., girls have a higher likelihood to nominate friends than boys), incoming nominations (e.g., girls have a higher likelihood to receive friendship nominations than boys), and nominations of similar others (“similarity”; e.g., girls have a higher likelihood to nominate girls and boys to nominate boys). The classes in our data consisted of only 12–20 pupils, making it impossible to estimate the ERGMs separately per class. We therefore created a blocked adjacency matrix that comprised all ten classes combined and used the block‐constraint function in the ERGM package to restrict ties to only exist between early adolescents in the same class while simultaneously allowing parallel simulation (Caravita et al. 2014; Kim et al. 2024).

Share of missing network data per class ranged from 0% to 27.27% (only two networks with > 20% missing network data; 8.2% of the eligible sample did not participate). Our missingness is therefore within the range of missing data levels that can be handled by social network analyses as indicated by previous research (De la Haye et al. 2017; Krause et al. 2020). Given that the share of missingness was acceptable and that we only had data available from 10 classes, we decided to keep all classes in. Early adolescents who were missing made no nominations themselves, but could still be nominated by their peers. Given that ERGMs cannot handle missing data in attributes (Rawlings et al. 2023), we replaced the missing values in early adolescents' acculturation orientations and control variables with the mean and mode of the respective variables (7.5%–12.6% of the eligible sample across variables). Given the relatively small sample size and number of classes, we cautiously interpreted 0.05 < *p* < 0.07 (Shi et al. 2022). We opted for *p* < 0.07 instead of the more conventional threshold of *p* < 0.10 for marginal effects, since effects with 0.07 < *p* < 0.10 were inconsistent across models (see also SOM.S1).

Following prior work (Smith et al. 2016), we controlled for several known structural network effects in school classes. Specifically, we controlled for the density of the network (i.e., the number of existing ties in the network relative to the number of possible ties in the network), reciprocity (i.e., the tendency to befriend someone who also nominates you), and transitivity (using “gwesp”; i.e., the tendency to befriend a friend of your friend). The goodness of fit increased with increasing decay values of the transitivity effect. Higher decay values imply that the probability of a friendship increases regardless of the number of shared friends two early adolescents have (Rawlings et al. 2023). Lower decay values imply that the probability of a friendship does not increase much if two early adolescents already share some friends (Rawlings et al. 2023). Higher decay values therefore allow the model more freedom to close triads of early adolescents (i.e., a friend of a friend is my friend) compared to lower decay values. This is often more beneficial in denser networks like the class networks in our study. We used a decay value of 0.5 in the final model as it is the optimal maximum value in school classes (Goodreau 2007). Yet, we replicated the final results using a decay value of 0.25 and 0.85 as a robustness check (see SOM.S1). Results remained the same. Finally, we also controlled for the logarithm of class size to account for the different number of peers that early adolescents in different classes could nominate (Krivitsky et al. 2011). The study's design and its analyses were not preregistered, but the R code and data are available on OSF (https://osf.io/4aqtj/overview?view_only=cb8213666ad24199a07ab0462f42a1f0). We reported all measures, analyses, and data handling transparently.

## 3 Results

Descriptive statistics can be found in Table 1. Early adolescents reported on average 4.51 friends (SD = 3.33, range 0–18). Although heritage cultural behaviors and identities were on average somewhat stronger than mainstream cultural behaviors and identities, early adolescents reported moderate to strongly orientations toward both cultures (although with quite some variation). Early adolescents reported rather similar average levels of heritage cultural behaviors and heritage cultural identities; and also rather similar average levels of mainstream cultural behaviors and mainstream cultural identities. This was in line with the strong correlations between heritage cultural behaviors and heritage cultural identities, and between mainstream cultural behaviors and mainstream cultural identities, respectively. Interestingly, none of the correlations between both cultures were significant. Given the strong correlations between behaviors and identities within cultures, we estimated our final model separately for behaviors and identities as a robustness check (see SOM.S2). Results were largely the same.

**Table 1:** Descriptive statistics.

	1	2	3	4	5	6	7	8
1 Age	—							
2 Girls (vs. boys)	−0.17*	—						
3 Moroccan origin (vs. non‐Moroccan origin)	−0.03	0.13	—					
4 Non‐Muslim (vs. Muslim)	0.17	−0.09	−0.15	—				
5 Mainstream culture behavior	0.15	−0.06	−0.21*	−0.03	—			
6 Heritage culture behavior	0.00	0.07	0.06	0.05	−0.01	—		
7 Mainstream culture identity	0.20*	0.09	−0.09	0.02	0.46***	0.01	—	
8 Heritage culture identity	0.18*	0.05	0.10	0.01	0.04	0.66***	0.11	—
*M*/%	11.24	45.6%	68.0%	11.5%	3.16	4.39	3.33	4.35
SD	0.83				0.94	0.74	1.17	0.81
Range	10–13				1–5	2–5	1–5	1–5

* *p* < 0.05;

*** *p* < 0.001.

### 3.1 Associations Between Cultural Behaviors and Identities and Friendship Networks

The full results can be found in Table 2 (see SOM.S3 for the goodness of fit of the model). In terms of *structural effects*, we found significant effects of density, reciprocity, transitivity, and class size. Density should be interpreted as an intercept in ERGMs; its significant negative value indicates that it is unlikely that friendship ties are formed at random and that friendship networks were rather sparse (Leszczensky et al. 2016; Smith et al. 2016). Moreover, we found positive effects of reciprocity and transitivity, indicating that early adolescents are more likely to nominate peers who have also nominated them as friends and that they are more likely to nominate friends of their friends. The negative effect of class size indicated that friendship ties are less likely in larger school classes.

**Table 2:** Predicting friendship ties from cultural behaviors and identities.

			*B*	SE	*p*
Structural effects	Density		−2.74	0.83	< 0.001***
	Reciprocity		1.52	0.21	< 0.001***
	Transitivity (gwesp)		1.18	0.09	< 0.001***
	Class size (log)		−0.82	0.17	< 0.001***
Controls	Age	Outgoing	−0.00	0.08	0.960
		Incoming	−0.01	0.08	0.942
		Similarity	−0.06	0.06	0.325
	Girl (vs. boy)	Outgoing	−0.36	0.12	0.003**
		Incoming	0.41	0.12	< 0.001***
		Similarity	1.12	0.08	< 0.001***
	Moroccan origin	Outgoing	0.17	0.13	0.184
	(vs. non‐Moroccan)	Incoming	−0.17	0.13	0.192
		Similarity	0.19	0.10	0.062+
	Non‐Muslim	Outgoing	0.76	0.26	0.004**
	(vs. Muslim)	Incoming	0.56	0.26	0.027*
		Similarity	0.87	0.23	< 0.001***
Main effects	Mainstream behavior	Outgoing	0.22	0.07	0.002**
		Incoming	−0.18	0.07	0.008**
		Similarity	0.04	0.05	0.447
	Heritage behavior	Outgoing	0.06	0.11	0.592
		Incoming	−0.11	0.11	0.330
		Similarity	−0.17	0.09	0.058+
	Mainstream identity	Outgoing	−0.16	0.05	0.005**
		Incoming	0.09	0.05	0.096
		Similarity	−0.02	0.05	0.636
	Heritage identity	Outgoing	0.20	0.10	0.060+
		Incoming	−0.12	0.11	0.254
		Similarity	0.03	0.09	0.740

*Note*. For categorical variables, similarity means friendship ties are predicted from sharing the same attribute. For continuous variables, similarity means friendship ties are predicted from the absolute difference in the attribute.

^+^ *p* < 0.07;

* *p* < 0.05;

** *p* < 0.01;

*** *p* < 0.001.

In terms of *control variables*, we found significant similarity effects of gender, Moroccan origin, and religion. We did not find effects of age. Specifically, we found that early adolescents were more often friends with peers with the same gender, that Moroccan‐origin early adolescents were more often friends with other Moroccan‐origin peers (and thus also that non‐Moroccan‐origin early adolescents were *less* often friends with Moroccan‐origin peers and *more* often with non‐Moroccan‐origin peers), and that Muslim early adolescents more often had friendships with other Muslims (and non‐Muslims *less* often with Muslim peers and *more* often with non‐Muslim peers). We therefore showed early adolescents' preference for peers with the same gender, ethnicity, and religion.

Importantly, cultural behaviors and identities were significantly associated with early adolescents' friendship ties in minority‐only schools over and beyond these effects. For *heritage cultural behaviors*, early adolescents were more likely to have friendships with peers who had similar levels of heritage cultural behaviors (similarity effect). Moreover, early adolescents with stronger *heritage cultural identities* reported more friendships than peers with weaker heritage cultural identities (outgoing effect). In short, early adolescents' heritage cultures were therefore associated with more friendships with peers in minority‐only schools, both in terms of heritage cultural behaviors and identities.

Yet, we find different patterns regarding the mainstream culture. In line with the findings for the heritage culture, early adolescents who reported higher levels of *mainstream cultural behaviors* were more likely to report more friendships compared to peers with lower levels of mainstream cultural behaviors (outgoing effect). However, at the same time, these early adolescents were also less likely to be nominated as a friend by others (incoming effect). Early adolescents with higher levels of mainstream cultural behaviors were thus more likely to nominate peers as their friends, but also less likely to receive a nomination from their peers. Additionally, early adolescents with stronger *mainstream cultural identities* reported fewer friendships than peers with weaker mainstream cultural identities (outgoing effect), so that stronger mainstream cultural identities were associated with fewer nominated friends.

## 4 Discussion

The present study adds to an emerging line of research examining the association between adolescents' acculturation orientations and their friendship networks. By combining an intergroup relations approach of acculturation (Brown and Zagefka 2011; Phalet and Baysu 2020) with an ecological‐systems theory approach (Bronfenbrenner 2005), we conceive of acculturation processes as part of adolescent development in the peer context. We extend prior work by distinguishing between cultural identities and cultural behaviors as separate acculturation domains (Schwartz et al. 2010) and linking them to minoritized youth's friendship ties. We also add to the current knowledge by zooming in on minority‐only schools as contexts that are bypassed in acculturation research. Using sociometric data of early adolescents' friendship networks in minority‐only school classes in Molenbeek, Brussels, Belgium, we found that acculturation orientations were associated with minoritized early adolescents' friendship ties over and beyond known factors like gender, ethnicity, and religion. Moreover, we demonstrated that cultural identities and cultural behaviors had different associations with friendship ties, therefore showing distinct effects of separate acculturation domains.

### 4.1 Acculturation Orientations and Friendship Networks in Minority‐Only Schools

Our hypothesis that stronger mainstream and heritage cultural orientations would be associated with more friendships in minority‐only school classes was partially confirmed. In agreement with our hypothesis, heritage cultural orientations were indeed associated with more friendship ties. Specifically, early adolescents with more similar levels of heritage cultural behaviors were more likely to be friends with each other; and early adolescents with stronger heritage cultural identities nominated more friends in class. We extend on prior work that showed influence (Jugert et al. 2020; Rivas‐Drake et al. 2017) and selection (Leszczensky and Pink 2019) processes in friendship networks in the domain of heritage cultural identities. Specifically, we demonstrate that similar processes might be at play for heritage cultural behaviors; and that similar processes take place in minority‐only schools as in schools with a different ethnic composition. Arguably, heritage cultural identities and behaviors are critical for early adolescents' friendships in minority‐only schools because everyone has a distinct heritage culture. It is likely that heritage cultural orientations are accordingly perceived as valuable and are promoted among peers in minority‐only schools (Hillekens et al. 2019; Rock et al. 2011). Aligning with this norm thus seems to benefit youth to be more embedded within friendship networks (Celeste et al. 2016).

However, the story is a little more nuanced when it comes to mainstream cultural orientations. In line with our hypothesis, mainstream cultural behaviors were associated with more (perceived) friendships in minority‐only schools. Specifically, early adolescents who reported more mainstream cultural behaviors nominated more friends in class. Yet, they were also less likely to be nominated by their peers as their friend. It thus seems that mainstream cultural behaviors are associated with more *perceived* friendships, despite it not being reciprocated by peers. Belgian schools generally have a strong emphasis on the mainstream culture and expect pupils to behave in line with the mainstream culture, such as by speaking Dutch (Celeste et al. 2019). Consequently, some early adolescents might perceive mainstream cultural behaviors as the norm in school. We speculate that they might think that adhering to this perceived norm also renders more friendships with peers. At the same time, their peers might not value the mainstream culture as much so that the actual norm is different, resulting in friendships not being reciprocated.

This also aligns with our findings regarding mainstream cultural identities. Contrary to our hypothesis, early adolescents with stronger mainstream cultural identities nominated *fewer* friends in class (i.e., regardless of the mainstream cultural identity of their peers). This is contrary to the study by Jugert et al. (2017) who showed that minoritized adolescents were more often friends with other minoritized peers with similar mainstream cultural identities. Such a pattern would have been represented by a significant similarity effect in our data, which did not appear. However, the study by Jugert et al. (2017) did not include separate measures for heritage and mainstream cultural identities, did not include cultural behaviors, and was conducted in schools including (more) ethnic majoritized peers. Potentially, effects might differ in minority‐only schools as highly segregated acculturation contexts. Importantly, mainstream cultural identities were moderate on average, but showed a lot of variation. It is therefore possible that the mainstream culture might not be as valued in some classes in our study, so that early adolescents with strong mainstream cultural identities do not fit in with the norm in the class.

Specifically, strong mainstream cultural identities are sometimes perceived as disloyalty toward the ethnic minoritized group, whereas mainstream cultural behaviors are not seen as such (Van Laar et al. 2014). This also aligns with prior work showing that belonging to the majoritized group might come at the cost of minoritized group belonging (Kende et al. 2020). Consequently, it might be that early adolescents experience stronger backlash from peers on their mainstream cultural identities than behaviors. In line with identity homophily theory (Stets et al. 2021), people prefer to cease interactions with others who do not verify their identities. However, it might be hard for early adolescents to stop interacting with peers in class who do not support their identities as they see these peers on a daily basis. It might therefore be that they are forced to continue interacting with peers whose acculturation norm is inconsistent with their mainstream identity, leaving them at risk for peer exclusion (Celeste et al. 2016) as well as more negative emotions and lower well‐being (Stets et al. 2021). Although it was beyond the scope of the present study, it might be that negative associations between mainstream cultural identities and early adolescents' friendships are therefore dependent on specific peer norms in class (Celeste et al. 2016). It is therefore critical to study when and how mainstream cultural identities (and behaviors) could be linked to more friendships in order to promote the well‐being of early adolescents in minority‐only schools.

Interestingly, we observed additional domain‐specific patterns in the association between acculturation orientations and early adolescents' friendships. Specifically, whereas cultural *behaviors* were also linked to incoming and similarity nominations by peers, cultural *identities* were only associated with outgoing nominations. Identities are internal states that signal cultural attachments, whereas behaviors relate to cultural performances. Accordingly, identities are less visible to others and might consequently only be linked to outgoing nominations. Behaviors are more overt and hence observable by peers, and might consequently also be linked to incoming and similarity nominations by peers. In line with identity homophily theory (Stets et al. 2021), identities drive our choices of how we interact with others, and behaviors verify our identities and ensure that they align with contextual expectations. Although this idea was supported by strong correlations between cultural identities and behaviors, it is important to further examine the interplay between cultural behaviors and identities and early adolescents' friendships in minority‐only schools. Our findings combined showed differential associations between cultural behaviors and identities and early adolescents' friendships, calling for a more domain‐specific approach toward acculturation.

### 4.2 Limitations and Future Directions

There are also some limitations that should be mentioned. First, we asked early adolescents to select the cultural group they felt most belonging to besides Belgian when assessing heritage cultural behaviors and identities. However, many early adolescents selected more than one group (although this was not as intended), with some even selecting up to six different cultural groups. The high internal consistencies of most of the behavior and identity measures suggest that most early adolescents had a clear idea about their (heritage) cultural behaviors and identities. Yet, this raises the question how early adolescents reflected upon their heritage culture and whether bidimensional models of acculturation are accurate in the context of minority‐only schools and beyond. This highlights that we might need to move toward measures that can capture hybrid or intersecting cultural identities (e.g., Turkish–Belgian) and that align more closely with the lived experiences of minoritized youth in how we measure cultural behaviors and identities. It also sparks questions like how early adolescents come to select certain cultural groups and whether this is co‐constructed among peers, especially when they select many like some early adolescents in the present study. For example, it might be that they start to feel a little Italian when they have many Italian friends. Future research should shed more light on early adolescents' self‐categorizations and how they emerge and are co‐constructed among peers, specifically in minority‐only schools.

Relatedly, the internal consistency of mainstream cultural behaviors was relatively low. We focused specifically on items examining whether early adolescents liked to do things together with children with a Belgian background, celebrate Belgian holidays, and eat Belgian food. It is likely that these behaviors do not show a consistent pattern in minority‐only contexts. For example, Belgian holidays are often also Christian holidays, whereas the majority of the early adolescents in our study were Muslims. Similarly, it might be that they have little opportunity to interact with ethnic majoritized peers. It is therefore interesting to see that mainstream cultural behaviors were still associated with friendships in our study, but it might be important to examine which behaviors are specifically relevant in minority‐only schools. Future research may need to develop new measures examining different types of cultural behaviors and linking them to friendship networks in minority‐only schools.

Third, we could not model the interplay between acculturation orientations and ethnicity because the model was already very complex considering our small sample and number of classes. Moreover, the majority of our sample had a Moroccan origin and were Muslims. Although we controlled for ethnic and religious homophily, it might be that acculturation orientations show stronger associations with co‐ethnic than cross‐ethnic friendships, because they share the same heritage culture (Jugert et al. 2020; Leszczensky and Pink 2019). This effect might be even stronger when different socialization contexts reinforce each other, for example, when friends in the neighborhood and friends in school display similar cultural expectations (Repke and Benet‐Martínez 2017). It might be worthwhile to examine interplays between acculturation orientations and ethnicity as well as between different acculturation contexts to shed further light on how we can promote early adolescents' friendships in minority‐only schools.

Finally, our cross‐sectional design prevented us from drawing any temporal conclusions, so that the direction of the associations between acculturation orientations and friendships remains unclear. Prior longitudinal work has shown that processes might be bidirectional (Jugert et al. 2017, 2020; Leszczensky et al. 2016; Leszczensky and Pink 2019; Munniksma et al. 2015; Rivas‐Drake et al. 2017), so that cultural behaviors and identities are formed by, but also shape friendship networks in schools. Still, these studies did not focus on minority‐only schools where unique processes might be at play. It therefore seems relevant to invest in large‐scale, longitudinal studies in minority‐only schools in different neighborhoods to shed further light on the processes under investigation in the present study.

## 5 Conclusion

Strong ethnic segregation in European societies has led to growing numbers of minority‐only schools. Although many minoritized adolescents reside in these schools, we know little about their acculturation orientations and their associations with friendship networks. Using sociometric data on friendship networks in minority‐only school classes in Molenbeek, Brussels, Belgium, this study examined whether early adolescents' acculturation orientations would be associated with their friendship ties; and explored cultural identities and behaviors as separate acculturation domains. We found that heritage cultural identities and behaviors were associated with more friendship ties. However, whereas mainstream cultural behaviors were associated with *more* perceived inclusion, mainstream cultural identities were associated with *less* friendship ties in minority‐only schools. Our study calls for a more domain‐specific approach toward acculturation as well as more research examining acculturation and development in minority‐only schools. Our findings highlight the critical role of acculturation orientations in minoritized early adolescents' friendships in minority‐only schools as a promising route to promote their sociocultural adjustment.

## Ethics Statement

All procedures performed in studies involving human participants were in accordance with the ethical standards of the institutional and/or national research committee and with the 1964 Helsinki Declaration and its later amendments or comparable ethical standards. The study was approved by the Social and Societal Ethics Committee of the University of Leuven.

## Conflicts of Interest

The authors declare no conflicts of interest.

## Supporting information


**Table S1:** Predicting friendship ties from cultural behaviors and identities using a decay value of 0.25. **Table S2:** Predicting friendship ties from cultural behaviors and identities using a decay value of 0.85. **Table S3:** Predicting friendship ties from cultural behaviors separately. **Table S4:** Predicting friendship ties from cultural identities separately. **Figure S1:** Goodness of fit of the model with a decay value of 0.25. **Figure S2:** Goodness of fit of the model with a decay value of 0.85. **Figure S3:** Goodness of fit of the final model as reported in the main paper.

## Data Availability

The data sets and R code generated and analyzed during the current study are publicly available via the following link: https://osf.io/4aqtj/overview?view_only=cb8213666ad24199a07ab0462f42a1f0.

## Endnotes

1 Unfortunately, we did not provide children with nonbinary gender options at the time of the data collection.
