# Supplementary material for: To Be Bicultural Is to Be Friends? Associating Early Adolescents' Cultural Identities and Behaviors With Friendship Networks in Minority‐Only Schools
Source: J Community Psychol. 2026 Jan 7;54(1):e70081. doi: 10.1002/jcop.70081 (PMC12778897; doi:10.1002/jcop.70081)
Supplement: Supplementary file 1 — Table S1: Predicting friendship ties from cultural behaviors and identities using a decay value of 0.25. Table S2: Predicting friendship ties from cultural behaviors and identities using a decay value of 0.85. Table S3: Predicting friendship ties from cultural behaviors separately. Table S4: Predicting friendship ties from cultural identities separately. Figure S1: Goodness of fit of the model with a decay value of 0.25. Figure S2: Goodness of fit of the model with a decay value of 0.85. Figure S3: Goodness of fit of the final model as reported in the main paper. [file JCOP-54-0-s001.docx]

**Supplementary online materials**

**SOM.1: Robustness check using different decay values**

We tested the final model using different decay values. Specifically, the final model in the paper had a decay value of 0.5, but we replicated this model with a decay value of 0.25 and 0.85, given that goodness of fit increased with increasing decay values (Figures S1 and S2). As can be seen in Tables S1 and S2, results remained stable regardless.

**Table S1.**

*Predicting friendship ties from cultural behaviors and identities using a decay value of 0.25.*

|  |  |  | | *B* | *SE* | *p* |
| --- | --- | --- | --- | --- | --- | --- |
| Structural effects | Density | |  | -2.76 | 0.98 | .005** |
|  | Reciprocity | |  | 1.71 | 0.19 | <.001*** |
|  | Transitivity (gwesp) | |  | 1.49 | 0.15 | <.001*** |
|  | Class size (log) | |  | -0.78 | 0.20 | <.001*** |
| Controls | Age | | Outgoing | -0.01 | 0.08 | .932 |
|  |  | | Incoming | -0.02 | 0.09 | .815 |
|  |  | | Similarity | -0.06 | 0.07 | .378 |
|  | Girl (vs. boy) | | Outgoing | -0.44 | 0.12 | <.001*** |
|  |  | | Incoming | 0.35 | 0.12 | .005** |
|  |  | | Similarity | 1.16 | 0.09 | <.001*** |
|  | Moroccan origin | | Outgoing | 0.19 | 0.14 | .160 |
|  | (vs. non-Moroccan) | | Incoming | -0.14 | 0.13 | .281 |
|  |  | | Similarity | 0.20 | 0.10 | .055+ |
|  | Non-Muslim | | Outgoing | 0.75 | 0.26 | .004** |
|  | (vs. Muslim) | | Incoming | 0.55 | 0.26 | .031* |
|  |  | | Similarity | 0.85 | 0.23 | <.001*** |
| Main effects | Mainstream behavior | | Outgoing | 0.22 | 0.07 | .002** |
|  |  | | Incoming | -0.18 | 0.07 | .012* |
|  |  | | Similarity | 0.03 | 0.05 | .628 |
|  | Heritage behavior | | Outgoing | 0.06 | 0.12 | .601 |
|  |  | | Incoming | -0.13 | 0.12 | .280 |
|  |  | | Similarity | -0.18 | 0.09 | .043* |
|  | Mainstream identity | | Outgoing | -0.16 | 0.06 | .004** |
|  |  | | Incoming | 0.09 | 0.06 | .108 |
|  |  | | Similarity | -0.01 | 0.05 | .887 |
|  | Heritage identity | | Outgoing | 0.22 | 0.11 | .043* |
|  |  | | Incoming | -0.10 | 0.11 | .353 |
|  |  | | Similarity | 0.04 | 0.09 | .637 |

*+ p* < .07, ** p* < .05, ** *p* < .01, *** *p* < .001

*Note.* For categorical variables, similarity means friendship ties are predicted from sharing the same attribute. For continuous variables, similarity means friendship ties are predicted from the absolute difference in the attribute.

**Figure S1.**

*Goodness of fit of the model with a decay value of 0.25.*
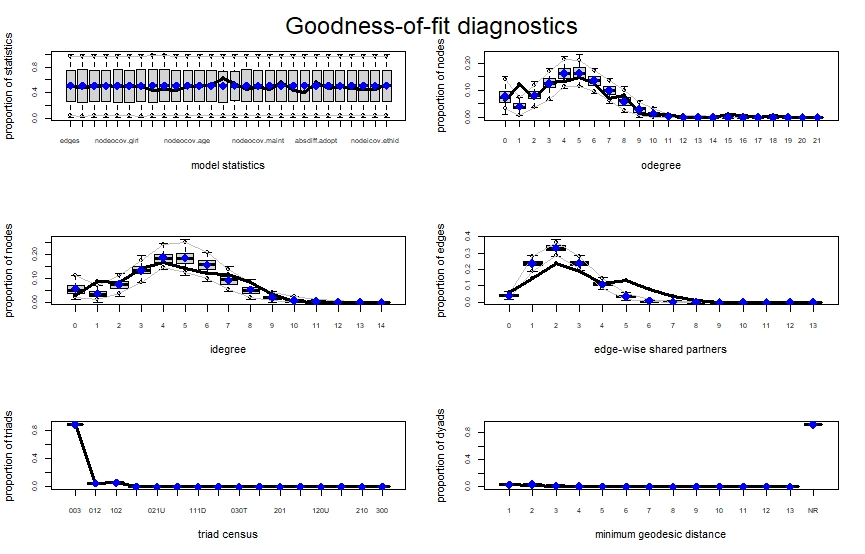


**Table S2.**

*Predicting friendship ties from cultural behaviors and identities using a decay value of 0.85.*

|  |  |  | | *B* | *SE* | *p* |
| --- | --- | --- | --- | --- | --- | --- |
| Structural effects | Density | |  | -2.38 | 0.74 | .001** |
|  | Reciprocity | |  | 1.39 | 0.21 | <.001*** |
|  | Transitivity (gwesp) | |  | 0.75 | 0.05 | <.001*** |
|  | Class size (log) | |  | -0.83 | 0.13 | <.001*** |
| Controls | Age | | Outgoing | -0.01 | 0.08 | .896 |
|  |  | | Incoming | 0.00 | 0.08 | .955 |
|  |  | | Similarity | -0.06 | 0.06 | .331 |
|  | Girl (vs. boy) | | Outgoing | -0.34 | 0.12 | .004** |
|  |  | | Incoming | 0.44 | 0.12 | <.001*** |
|  |  | | Similarity | 1.17 | 0.08 | <.001*** |
|  | Moroccan origin | | Outgoing | 0.16 | 0.13 | .235 |
|  | (vs. non-Moroccan) | | Incoming | -0.19 | 0.13 | .143 |
|  |  | | Similarity | 0.19 | 0.11 | .067+ |
|  | Non-Muslim | | Outgoing | 0.77 | 0.27 | .005** |
|  | (vs. Muslim) | | Incoming | 0.59 | 0.27 | .026* |
|  |  | | Similarity | 0.91 | 0.24 | <.001*** |
| Main effects | Mainstream behavior | | Outgoing | 0.22 | 0.07 | .002** |
|  |  | | Incoming | -0.19 | 0.07 | .009** |
|  |  | | Similarity | 0.06 | 0.05 | .206 |
|  | Heritage behavior | | Outgoing | 0.04 | 0.11 | .732 |
|  |  | | Incoming | -0.10 | 0.11 | .380 |
|  |  | | Similarity | -0.19 | 0.09 | .044* |
|  | Mainstream identity | | Outgoing | -0.16 | 0.05 | .004** |
|  |  | | Incoming | 0.09 | 0.05 | .108 |
|  |  | | Similarity | -0.05 | 0.05 | .331 |
|  | Heritage identity | | Outgoing | 0.20 | 0.10 | .054+ |
|  |  | | Incoming | -0.12 | 0.11 | .239 |
|  |  | | Similarity | 0.04 | 0.09 | .662 |

*+ p* < .07, ** p* < .05, ** *p* < .01, *** *p* < .001

*Note.* For categorical variables, similarity means friendship ties are predicted from sharing the same attribute. For continuous variables, similarity means friendship ties are predicted from the absolute difference in the attribute.

**Figure S2.**

*Goodness of fit of the model with a decay value of 0.85.*


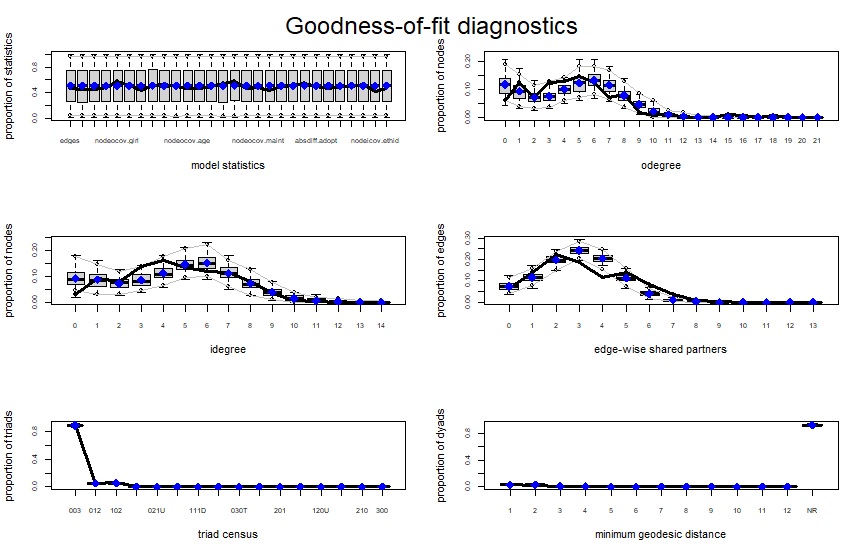


**SOM.2: Robustness check estimating separate models for behaviors and identities**

Given the strong correlations between cultural behaviors and cultural identities, we tested the final model separately for both acculturation domains. As can be seen in Tables S3 and S4, the negative association between mainstream identities and adolescents’ outgoing friendship nominations was only significant when taking their cultural behaviors into account. Additionally, a negative effect of heritage cultural identities on incoming friendship nominations emerged when not taking cultural behaviors into account. All other effects replicated when testing separate models for cultural behaviors and identities.

**Table S3.**

*Predicting friendship ties from cultural behaviors separately.*

|  |  |  | | *B* | *SE* | *p* |
| --- | --- | --- | --- | --- | --- | --- |
| Structural | Density | |  | -2.42 | 0.79 | .002** |
| effects | Reciprocity | |  | 1.47 | 0.20 | <.001*** |
|  | Transitivity (gwesp) | |  | 1.18 | 0.09 | <.001*** |
|  | Class size (log) | |  | -0.82 | 0.16 | <.001*** |
| Controls | Age | | Outgoing | 0.01 | 0.08 | .871 |
|  |  | | Incoming | -0.05 | 0.08 | .524 |
|  |  | | Similarity | -0.05 | 0.06 | .419 |
|  | Girl (vs. boy) | | Outgoing | -0.39 | 0.12 | <.001*** |
|  |  | | Incoming | 0.42 | 0.12 | <.001*** |
|  |  | | Similarity | 1.12 | 0.08 | <.001*** |
|  | Moroccan origin | | Outgoing | 0.17 | 0.13 | .198 |
|  | (vs. non-Moroccan) | | Incoming | -0.18 | 0.13 | .170 |
|  |  | | Similarity | 0.19 | 0.10 | .066+ |
|  | Non-Muslim | | Outgoing | 0.73 | 0.26 | .005** |
|  | (vs. Muslim) | | Incoming | 0.61 | 0.25 | .016* |
|  |  | | Similarity | 0.88 | 0.23 | <.001*** |
| Main effects | Mainstream behavior | | Outgoing | 0.14 | 0.06 | .027* |
|  |  | | Incoming | -0.15 | 0.06 | .017* |
|  |  | | Similarity | 0.03 | 0.05 | .501 |
|  | Heritage behavior | | Outgoing | 0.20 | 0.08 | .014* |
|  |  | | Incoming | -0.21 | 0.09 | .014* |
|  |  | | Similarity | -0.15 | 0.08 | .066+ |

*+ p* < .07, ** p* < .05, ** *p* < .01, *** *p* < .001

*Note.* For categorical variables, similarity means friendship ties are predicted from sharing the same attribute. For continuous variables, similarity means friendship ties are predicted from the absolute difference in the attribute.

**Table S4.**

*Predicting friendship ties from cultural identities separately.*

|  |  |  | | *B* | *SE* | *p* |
| --- | --- | --- | --- | --- | --- | --- |
| Structural | Density | |  | -2.58 | 0.82 | .002** |
| effects | Reciprocity | |  | 1.49 | 0.20 | <.001*** |
|  | Transitivity (gwesp) | |  | 1.17 | 0.09 | <.001*** |
|  | Class size (log) | |  | -0.85 | 0.16 | <.001*** |
| Controls | Age | | Outgoing | -0.02 | 0.08 | .844 |
|  |  | | Incoming | 0.00 | 0.08 | .996 |
|  |  | | Similarity | -0.06 | 0.06 | .358 |
|  | Girl (vs. boy) | | Outgoing | -0.37 | 0.12 | .002** |
|  |  | | Incoming | 0.46 | 0.12 | <.001*** |
|  |  | | Similarity | 1.12 | 0.08 | <.001*** |
|  | Moroccan origin | | Outgoing | 0.09 | 0.13 | .502 |
|  | (vs. non-Moroccan) | | Incoming | -0.11 | 0.13 | .373 |
|  |  | | Similarity | 0.20 | 0.10 | .046* |
|  | Non-Muslim | | Outgoing | 0.81 | 0.25 | .001** |
|  | (vs. Muslim) | | Incoming | 0.50 | 0.25 | .047* |
|  |  | | Similarity | 0.83 | 0.23 | <.001*** |
| Main effects | Mainstream identity | | Outgoing | -0.08 | 0.05 | .105 |
|  |  | | Incoming | 0.04 | 0.05 | .479 |
|  |  | | Similarity | -0.01 | 0.05 | .776 |
|  | Heritage identity | | Outgoing | 0.24 | 0.08 | .001** |
|  |  | | Incoming | -0.17 | 0.08 | .029* |
|  |  | | Similarity | -0.03 | 0.07 | .634 |

*+ p* < .07, ** p* < .05, ** *p* < .01, *** *p* < .001

*Note.* For categorical variables, similarity means friendship ties are predicted from sharing the same attribute. For continuous variables, similarity means friendship ties are predicted from the absolute difference in the attribute.

**SOM.3: Goodness of fit of the final model**

Figure S3 shows the goodness of fit of the final model. Goodness of fit of ERGM models is assessed by looking at the dark bold lines in the plots. Model fit is considered to be good when these lines stay between the lighter grey boundaries. The better the model fit, the more the dark bold lines will approach the midpoint between the grey boundaries and the more they follow a similar pattern. Figure S3 therefore shows that our model fitted the data well. Only the fit for the edge-wise shared partners can be considered to be acceptable, and further improved with larger decay values (see SOM.1). Although we opted for the model with a decay value of 0.5 (see the main paper), results were the same regardless. All other indicators showed good model fit.

**Figure S3.**

**
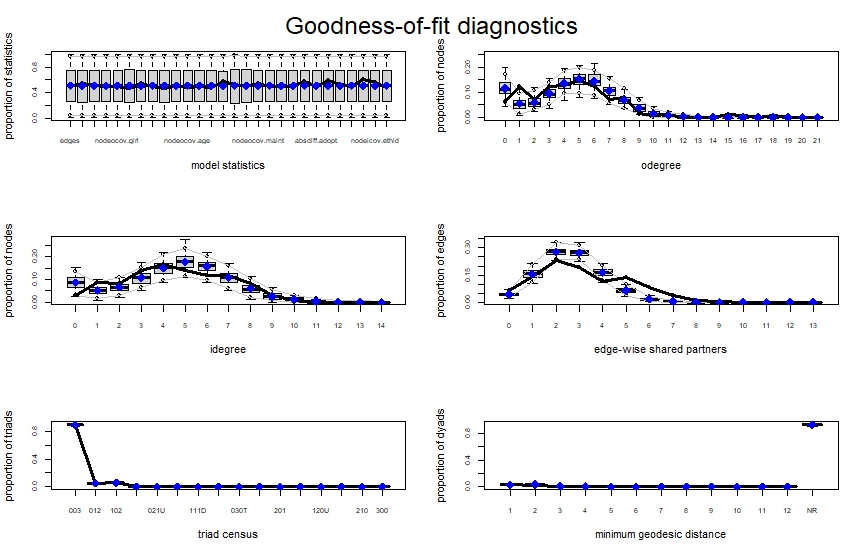
***Goodness of fit of the final model as reported in the main paper.*
